# Blind Abscess

**Published:** 1887-07

**Authors:** J. A. Thornton


ARTICLE V.

### "BLIND ABSCESS."

BY J. A. THORNTON, D. D. S.

[Read before the Georgia State Dental Society, May, 1887.]

To see is to know. But where the vision cannot penetrate, our success is wholly dependent upon experimental observations, which must of necessity be exercised with great care. Nor can we be too careful in anything we do, for our errors are here to stare not only us in the face, but to be commented upon by those whose knowledge consists in the amount of conceit usually attended with those who think they know it all, yet, when the test is applied, they only defend themselves with a child's logic, which is "because," and beyond because they can adduce no argument to sustain their severe criticisms upon those whose sole study and ardent desire is for the promotion of comfort and pleasure to those who are so unfortunate as to be forced to seek us for our skill. Nor are we always crowned with that degree of success for which we so ardently put forward our most earnest endeavors.

But were we to give up and say that on account of one failure it could not be accomplished, not only would we suffer defeat, but the hand of progress, which has so bountifully poured her plenty upon us, would be closed, and we, of necessity, be forced to see our profession, which we all love, lose its onward march and fall into the ruts of retrograde, and ere a few decades all the foundation of years of study and practical application, pass away and be forgotten. But we are not of that kind, and I thank God for it. But, on the other hand, when we fail it only renews our energies and makes us the more persistent to accomplish that success which always crowns diligent inquiry.

Now, as to my subject, perhaps there are some here who work and treat the trouble in the same manner as I; yet, as I have never read anything just like my own, I give it, and if it be that, or the same of some one of you here, perhaps there may be others to whom it may be of service.

Only one point in the following do I claim to belong entirely to some other than myself. Yet if there are others who have, as I have, been using their energies to conquer the most stubborn of our duties professionally, I only hope that he, or they, may have the coronet of success to fall upon him.

My own idea of Blind Abscess is where, at the apical foramen there is a sac containing pus without any fistulous opening, or any chance for discharge through the nares, or down the throat through the nasal channel.

It lies dormant until we, with our broaches, approach it, and then, like a little wasp, it shows how patiently it has waited for something to disturb it. Now for the approach, which I always accomplish by drilling directly to the nerve canal, being always careful to prevent any debris being lodged so as to be passed into the canal or through the apex, especially, for the presence of any foreign substance is sure to raise a huge disturbance, and that always retards our progress in effecting a cure, which is necessarily slow, as you never know what you have done until you have finished and the time for early trouble has passed.

After opening I make a solution of—

Tinct Iodine f℥ iij.
Aqua Ammonia f℥ j.

The iodine is antiseptic, has a tendency to prevent putrefaction and, also, is a disinfectant. It also acts as an escharotic without any tendency to discoloration. Unlike carbolic acid it does not coagulate the albumen and thereby form a stone foundation for acute inflammation, the termination of which is quite plain to all. The ammonia combines with the iodine and forms a transparent fluid, which, instead of discoloring the tooth, will tend to bleach it.

After having the tooth ready for treatment, I make of very fine grain hickory, a lot of broaches (wood is preferable because it is not affected by the solution like metal) around which I wind absorbent cotton and dip them, just as I use them, in the fluid, and the first I pass about half way to the apex to see if the canal is clear of debris. I then approach nearer and nearer, till I reach the foramen (apical), and I continue to apply till the last vestige of pus disappears, and dismiss my patient for twenty-four hours, leaving the tooth open and giving special directions to be careful to pack cotton loosely in the cavity during meals, but to remove as soon as the meal is finished.

The second sitting I pursue the same course and give the same directions. The third day, after having gone over first and second day's work in the same manner, with one of my broaches loosely wrapped with cotton and thoroughly saturated with the solution I pass it into the canal and dislodge the cotton loosely, so as to allow passage of gas, and if no soreness arises I allow it to remain for forty-eight hours. But should acute inflammation arise I remove the cotton at once and paint the gum over the root (camel's hair pencil) with a solution of iodine and aconite, equal parts, (not my own) and allow the tooth to rest till all traces of tenderness has passed, and then resort to the same means as first described for the same length of time and try again. However, with proper management, not one time in ten will any trouble arise. But now for the end which, after the third day, and you have closed the tooth loosely each sitting with fresh cotton, after having gone over third treatment, which should be at least from every forty-eight to seventy-two hours, till you can pack the canal tight and have no bad results. At which time saturate cotton with five per cent. solution carbolic acid and pack it as tightly as possible and allow it to remain, after filling with gutta percha, for a month, or even longer, then remove your filling, and also the cotton, and after one or two treatments with the carbolic acid you can then fill permanently and feel perfectly easy as to resuts.—*Southern Dental Journal.*

